# Striatal Dopamine Transmission Is Subtly Modified in Human A53Tα-Synuclein Overexpressing Mice

**DOI:** 10.1371/journal.pone.0036397

**Published:** 2012-05-03

**Authors:** Nicola J. Platt, Suzana Gispert, Georg Auburger, Stephanie J. Cragg

**Affiliations:** 1 Department of Physiology, Anatomy and Genetics, University of Oxford, Oxford, United Kingdom; 2 Department of Neurology, Goethe University Medical School, Frankfurt, Germany; University of Maryland School of Pharmacy, United States of America

## Abstract

Mutations in, or elevated dosage of, *SNCA*, the gene for α-synuclein (α-syn), cause familial Parkinson's disease (PD). Mouse lines overexpressing the mutant human A53Tα-syn may represent a model of early PD. They display progressive motor deficits, abnormal cellular accumulation of α-syn, and deficits in dopamine-dependent corticostriatal plasticity, which, in the absence of overt nigrostriatal degeneration, suggest there are age-related deficits in striatal dopamine (DA) signalling. In addition A53Tα-syn overexpression in cultured rodent neurons has been reported to inhibit transmitter release. Therefore here we have characterized for the first time DA release in the striatum of mice overexpressing human A53Tα-syn, and explored whether A53Tα-syn overexpression causes deficits in the release of DA. We used fast-scan cyclic voltammetry to detect DA release at carbon-fibre microelectrodes in acute striatal slices from two different lines of A53Tα-syn-overexpressing mice, at up to 24 months. In A53Tα-syn overexpressors, mean DA release evoked by a single stimulus pulse was not different from wild-types, in either dorsal striatum or nucleus accumbens. However the frequency responsiveness of DA release was slightly modified in A53Tα-syn overexpressors, and in particular showed slight deficiency when the confounding effects of striatal ACh acting at presynaptic nicotinic receptors (nAChRs) were antagonized. The re-release of DA was unmodified after single-pulse stimuli, but after prolonged stimulation trains, A53Tα-syn overexpressors showed enhanced recovery of DA release at old age, in keeping with elevated striatal DA content. In summary, A53Tα-syn overexpression in mice causes subtle changes in the regulation of DA release in the striatum. While modest, these modifications may indicate or contribute to striatal dysfunction.

## Introduction

Parkinson's disease (PD) is a neurodegenerative disorder characterized by the degeneration of nigrostriatal dopamine (DA) neurons, and the presence of proteinaceous aggregates, called Lewy bodies, in surviving DA neurons. A major component of Lewy bodies is a small protein called alpha synuclein (α-syn) [1]. Mutations in [2], [3], or elevated dosage of [4], *SNCA*, the gene coding for α-syn, including the A53T mutation [2], cause rare familial forms of PD in humans. Recent GWAS studies have also shown a strong association between single nucleotide polymorphisms (SNPs) in the *SNCA* locus and idiopathic PD [5], [6]. Therefore α-syn is strongly implicated in the pathogenesis of PD.

The protein α-syn is expressed presynaptically throughout the central nervous system but, despite extensive investigation [7]–[11], its exact functions remain incompletely resolved. Recent work has focused on its presynaptic roles in controlling vesicle fusion [11], [12], vesicle pool regulation [13]–[15] and vesicle clustering [16]. In cultured cells, overexpression of wild-type or mutant α-syn has been reported to cause deficits in vesicle cycling and transmitter release [11], [16]. However, the effect of mutant α-syn on dopaminergic signalling within the striatum, in regions differentially vulnerable to degeneration in PD, and in response to stimulation across the range of frequencies seen *in vivo*, remains unexplored.

Overexpression of different mutant *SNCA* has been used to generate several animal models of PD [17], [18]. Mouse lines overexpressing human A53Tα-syn under control of the mouse prion (PrP) promoter [19]–[22] constitute one such model. In these mice, the human α-syn transgene is expressed in neurons that include nigral neurons, and in striatal synapses, but not in striatal neurons [19]. These mice show pathological changes including abnormal subcellular localisation of α-syn in many brain regions, mild accumulation of insoluble α-syn in the substantia nigra and non-specific signs of neurodegeneration in the olfactory bulb, in the absence of overt nigrostriatal degeneration. They also show age-related motor deficits including reduced rearing, step length and rotarod performance. Furthermore, these mice show progressive presynaptic changes, including the upregulation of vesicle-associated proteins such as the chaperone 14-3-3 [21], and progressive striatal postsynaptic changes, including increased DA receptor expression, altered expression of proteins downstream of DA signalling and reduced corticostriatal long term depression [20], [22]. These changes are suggestive of progressive deficits in striatal DA signalling. Therefore these mice may model early PD-associated changes and hence are useful for investigating early pathological mechanisms.

Mutant α-syn has been shown to reduce transmitter release in cultured neurons [11], [16], and since A53Tα-syn overexpressing mice show changes suggestive of disrupted striatal DA transmission, we have directly investigated whether A53Tα-syn overexpression in mice causes deficits in dynamic striatal DA release. We have explored DA release in two independent lines of human A53Tα-syn overexpressing mice (lines A and B) compared to wild-type (WT) animals for two age ranges, 6–14 months and 18–24 months. We used fast-scan cyclic voltammetry (FCV) at carbon-fibre microelectrodes to detect DA release in real time, in acute striatal slices, in two striatal regions differentially affected in PD (caudate-putamen (CPu) and nucleus accumbens (NAc)).

## Methods

### Animals

Female mice overexpressing human A53Tα-syn under control of the mouse prion promoter, described previously [19], [20], were compared to wild-type mice (WT) of the same background strain, FVB/N. These A53Tα-syn overexpressing mice show an approximately 1.5 fold level of α-syn protein in the striatum, and high levels of α-syn expression in midbrain DA neuons [19], [20]. Mice from two different founder lines (line A and line B) were used, to control for effects due to random gene insertion. Mice were used at 2 ages, a mid-adult age (6–14 months), before the onset of motor impairments, and an old age (18–24 months) at which the onset of motor impairments has been reported [19]. All procedures were carried out in accordance with UK Home Office project licence and local guidelines.

### Slice preparation

Striatal slices were prepared as described previously [23], [24]. In brief, mice were sacrificed by cervical dislocation and the brains removed and transferred to ice-cold HEPES-based buffer containing in mM: 120 NaCl, 20 NaHCO_3_, 6.7 HEPES acid, 5 KCl, 3.3 HEPES salt, 2 CaCl_2_, 2 MgSO_4_, 1.2 KH_2_PO_4_, 10 glucose, saturated with 95%O_2_/5%CO_2_. Acute 300 µm thick coronal striatal slices, containing both dorsal striatum (CPu) and nucleus accumbens core (NAc) were prepared in ice-cold HEPES-based buffer and cut using a vibratome (VT1000S or VT1200S; Leica). Slices were kept at room temperature in HEPES-based buffer for 1 hour. Slices were transferred to the recording chamber and superfused at approximately 1.5 ml/min in bicarbonate buffer-based artificial CSF (aCSF) containing in mM: 124 NaCl, 26 NaHCO_3_, 3.8 KCl, 2.4 CaCl_2_, 1.3 MgSO_4_, 1.3 KH_2_PO_4_, 10 glucose, saturated with 95%O_2_/5%CO_2_, at 31–33°C. Slices were allowed to equilibrate for 30 minutes prior to recording.

### Electrical stimulation

DA release was evoked using a surface bipolar concentric Pt/Ir electode (25 µm diameter, FHC) as described previously [23], [24]. The stimulating electrode was placed locally, approximately 100 µm from the recording electrode. Stimulation pulses of 200 µs duration were applied at 0.6 mA, a perimaximal current (the minimum current found to evoke maximum DA release with a single 200 µs pulse).

### Voltammetry

Evoked extracellular DA concentration ([DA]_o_) was measured using FCV at carbon-fibre microelectrodes (diameter 7–10 µm, tip length 50–120 µm). A triangular voltage waveform was scanned across the microelectrode (−700 to +1300 mV and back vs Ag/AgCl reference, scan rate 800 V/s) using a Millar voltammeter, with a sampling frequency of 8 Hz, as described previously [24]. Evoked currents were confirmed as DA by comparison of the voltammogram with that produced during calibration with applied DA in aCSF (oxidation peak +500–600 mV and reduction peak −200 mV). Currents at the oxidation peak potential were measured from the baseline of each voltammogram and plotted against time to provide profiles of [DA]_o_ versus time. Electrodes were calibrated in 2 µM DA in aCSF. Calibration solutions were made immediately before use from stock solution of 2.5 mM DA in 0.1 M HClO_4_ stored at 4°C.

### Experimental Design and Data Analysis

Unless otherwise stated, repeated electrical stimuli were applied at 2.5 min intervals, by when release was reproducible and sustainable for several hours. [DA]_o_ varies between different striatal sampling sites [25]. To obtain representative measurements of DA release, [DA]_o_ was measured at 5 sites in CPu (dorsolateral, dorsomedial, ventrolateral, ventromedial and central) and 2 sites in NAc (lateral and medial), in each animal. Data was averaged within CPu and NAc, across animals. In each site, 2 single stimulus pulses, separated by 4 s were applied. [DA]_o_ evoked by the initial pulse was used as a measure of DA release, from a naive site. [DA]_o_ evoked by the second pulse was used as a measure of DA re-release following a single stimulus. In experiments investigating frequency response, data sets were obtained from single sites per animal, in dorsolateral CPu. Stimuli consisted of 4 pulses at a range of frequencies (5–100 Hz), covering the full range of DA neuron firing reported *in vivo* [26], [27]. Stimuli were given in a pseudorandomized order and in triplicate. In experiments investigating recovery of release following prolonged stimulation a single site per slice, in dorsolateral CPu, was used. A prolonged stimulation (10 Hz, 60 s) was applied and recovery of release monitored by a single stimulus pulse at 10, 30, 60, 120, 270 and subsequent 150 s intervals after the end of prolonged stimulation.

Data were acquired and analysed using Axoscope 10.2 (Molecular Devices) or Strathclyde Whole Cell Program (University of Strathclyde, Glasgow, UK) and locally written programs. Data are expressed as mean ± SEM with *n* = number of observations. The number of animals contributing to each data set was 3–8. To compare DA uptake in CPu, the falling phases of all DA profiles with a peak [DA]_o_ over 1.5 µM were time-locked to the timepoint when [DA]_o_ fell below 1.5 µM, averaged and compared between genotypes. This concentration of [DA]_o_ was selected to allow comparison of uptake at a concentration where signal to noise ratio is large, whilst still including a large number of data points. For experiments investigating frequency response, peak [DA]_o_ evoked by 4 pulses (5–100 Hz) was normalised to peak [DA]_o_ evoked by a single pulse in the same drug condition. In experiments investigating DA re-release following single pulse stimulation, peak [DA]_o_ evoked by a second pulse was normalised to peak [DA]_o_ evoked by the initial stimulation. In experiments investigating recovery of DA release following prolonged stimulation, data was normalised in two ways. To investigate the extent of recovery of release, data was normalised to peak [DA]_o_ evoked by the initial prolonged stimulation. To investigate the rate of recovery, data was normalised to the maximum recovery (calculated as the mean peak [DA]_o_ evoked by single pulse stimulations 35–42 mins following prolonged stimulation).

Comparisons for differences in means were carried out in GraphPad Prism using One or Two-way ANOVAs and *post hoc* Bonferroni's t tests. Curve fits were carried out in GraphPad Prism using nonlinear regression analysis, using sigmoidal dose-response (variable slope) or two-phase exponential analysis. Significance was considered as *P<0.05*.

### Drugs

D-AP5, Bicuculline methiodide, Dihydro-β-erythroidine (DHβE), 4-(8-methyl-9*H*-1,3-dioxolo[4,5-*h*][2], [3]benzodiazepin-5-yl)-benzenamine hydrochloride (GYKI 52466 hydrochloride), L-714,626, (S)-α-methyl-4-carboxyphenylglycine (MCPG) and saclofen were purchased from Tocris Biosciences or Ascent Scientific. Drugs were dissolved in distilled water or aqueous alkali ((S)-MCPG), aqueous acid (GYKI 52466 hydrochloride) or dimethylsulphoxide (DMSO) (L-741,626) to make stock aliquots 1000–10000× final concentrations and stored at −20°C. Final concentrations were made up in aCSF immediately before use.

## Results

### Single pulse-evoked dopamine release is not different in A53Tα-syn overexpressers

**Figure 1:**
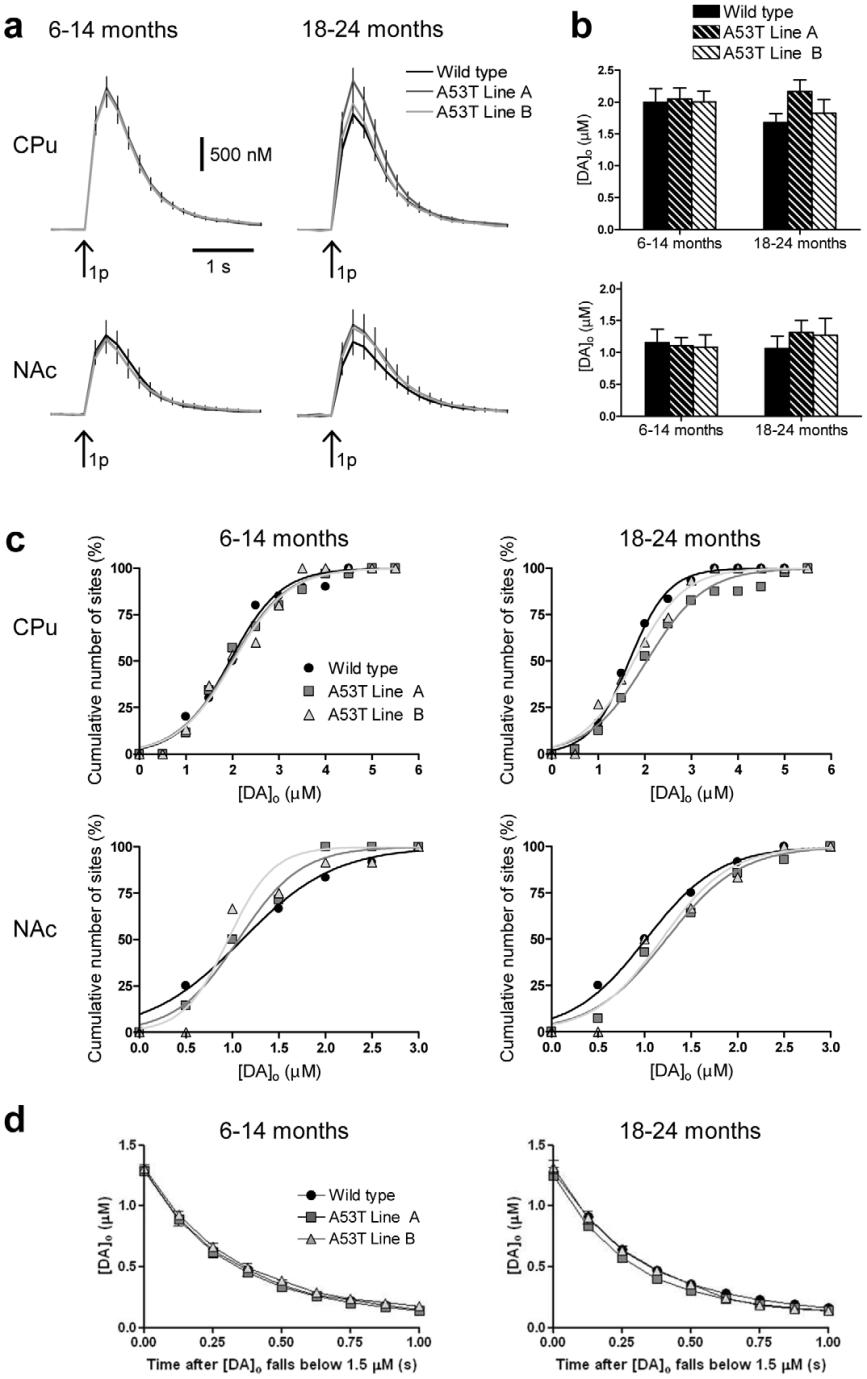
A53Tα-syn overexpression does not affect [DA]_o_ evoked by a single stimulus pulse. (**a**) Profiles of mean [DA]_o_± SEM versus time, after single pulse stimulation (arrow). There was no significant difference in [DA]_o_ between genotypes, at either age or in either region (*n = 15–40 (CPu), n = 6–14 (NAc)*). There was also no significant difference in mean peak [DA]_o_ between 6–14 and 18–24 month mice of any genotype (*n = 15–40 (CPu), n = 6–14 (NAc)*). (**b**) Mean peak [DA]_o_± SEM evoked by a single pulse. (**c**) Cumulative distribution plots of peak [DA]_o_ (µM) evoked by a single stimulation pulse. Curve fits are sigmoidal variable slope curves, *R^2^>0.93*. Curves are not significantly different between genotypes at 6–14 months in CPu or at either age in NAc, but are significantly different in CPu at 18–24 months. (**d**) Mean decay phases of concentration-matched DA transients in CPu do not differ between genotypes (*n* = 9–28).

FCV in acute striatal slices was used to investigate DA release in 2 lines of A53Tα-syn overexpressing mice (lines A and B) versus WT mice, at 2 ages, 6–14 months and 18–24 months. We first explored [DA]_o_ evoked by a single electrical stimulus pulse in each of two striatal regions, the caudate-putamen (CPu) and nucleus accumbens core (NAc). These regions are differentially affected in PD and DA release may be differentially controlled by synucleins in these regions [28]. Consistent with previous studies [25], [DA]_o_ was lower in NAc than in CPu in all genotypes (**Fig. 1a,b**). However there was no significant difference between genotypes in mean peak [DA]_o_ evoked by a single pulse, at either age, in either CPu (*n = 15–40*), or NAc (*n = 6–14*) (**Fig. 1a,b**). Data was additionally analysed by sub-region within CPu, and by appropriate groups of sub-regions (dorsal versus ventral, and medial versus lateral). There was no significant effect of genotype in any sub-region (*n = 3–8*), or any group of subregion (*n = 6–16*), at either age. Therefore data were grouped into a single CPu population for further analysis. There was also no significant difference in mean peak [DA]_o_ evoked by a single pulse between 6–14 month versus 18–24 month mice for any genotype, in either region (*n = 6–40*) (**Fig. 1a,b**). Cumulative distribution plots of evoked [DA]_o_ indicated broadly similar spreads of data in all genotypes, with the exception of in CPu in 18–24 month mice where distribution was significantly different between genotypes and the cumulative distribution plot showed a slightly decreased slope in A53Tα-syn overexpressors (**Fig. 1c**). The slopes of falling phases of DA transients are a function of DA uptake rate [29], and were compared between genotypes at a [DA]_o_ of 1.5 µM. There was no significant interaction between genotype and time after [DA]_o_ falling below 1.5 µM (*n* = 9–28) (**Fig. 1d**).

### A53Tα-syn overexpression modifies the frequency dependence of DA release

DA neurons fire at a range of frequencies *in vivo*, from tonic, low frequency, firing, at ∼2–5 Hz, to bursts of high frequency firing, at ∼15–40 Hz or higher [26], [27]. These firing patterns have different behavioural significance [30]. The filtering of these firing patterns by DA neuron terminals into DA release can be highly dynamic [24], [25], [31]. We investigated whether overexpression of A53Tα-syn differentially modified DA release in response to stimulations of different frequencies across this physiological range (4 pulses, 5–100 Hz). This was explored in dorsolateral CPu only.

**Figure 2:**
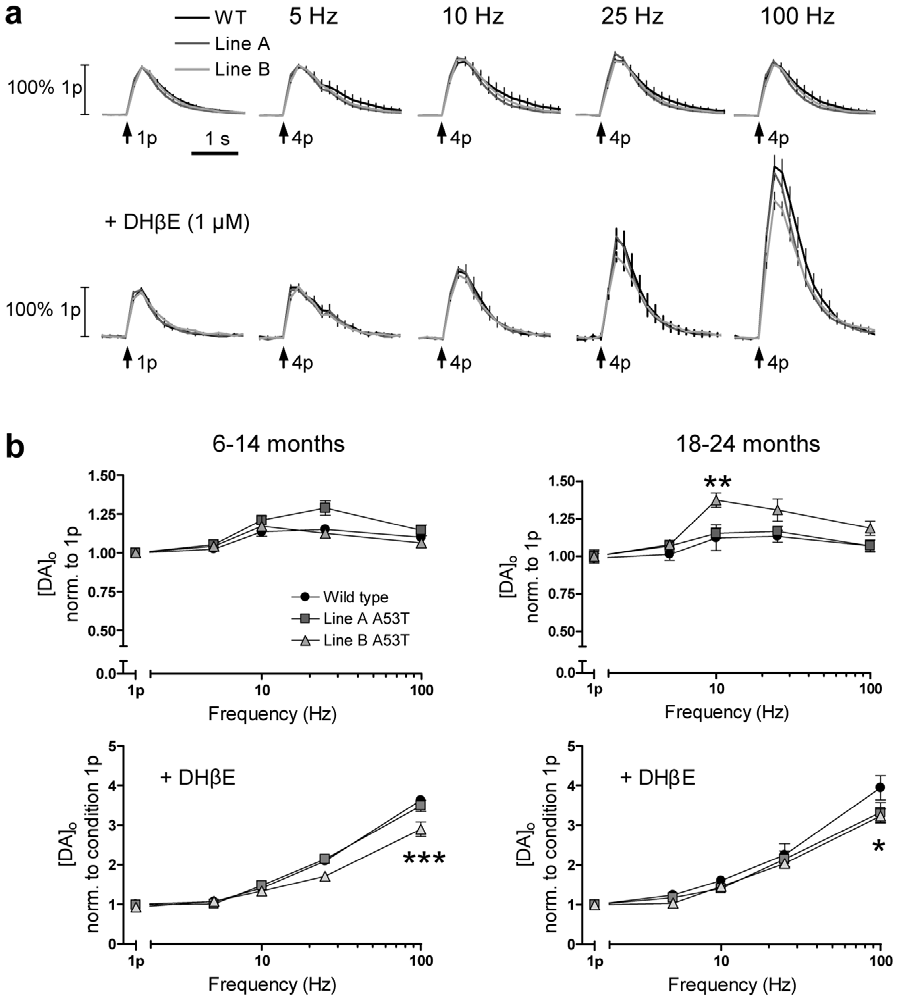
A53Tα-syn overexpression causes subtle deficits in DA release in response to high versus low frequency stimulation, when nAChRs are inhibited. (**a**) Profiles of mean [DA]_o_± SEM versus time, evoked by 4-pulse stimulation (1–100 Hz), with intact nAChR activity (upper), and with nAChR inhibition (DHβE, 1 µM) (lower)(6–14 months only shown). (**b**) Mean peak [DA]_o_±SEM evoked by 4-pulse stimulation (1–100 Hz), normalised to single pulse release, with nAChR activity (upper) and with nAChR inhibition (in DHβE, 1 µM)(lower). With intact nAChR activity, there was no significant difference in frequency response between genotypes at 6–14 months (*n = 9–15*). At 18–24 months, normalised peak [DA]_o_ evoked by 10 Hz stimulation was slightly greater in line B versus WT (2-way ANOVA, *post hoc* Bonferroni t tests, *p<0.05*; *n = 9–15*). With nAChR inhibition, in DHβE, at 6–14 months, normalised peak [DA]_o_ evoked by 100 Hz stimulation was significantly lower in line B than WT (2-way ANOVA, *post hoc* Bonferroni t test, *p<0.001*; *n = 9–15*) and, at 18–24 months, normalised peak [DA]_o_ evoked by 100 Hz stimulation was significantly lower in both A53Tα-syn overexpressing lines compared to WT (2-way ANOVA, *post hoc* Bonferroni t test, *p<0.05*; *n = 9–15*).

The frequency response of peak [DA]_o_ formed a characteristic inverted U shape [24], [32] in all genotypes, at both ages (**Fig. 2a,b** **upper**). This response is due to a number of activity-dependent processes acting on [DA]_o_, including strong short term depression of release [25], the activation of D_2_ autoreceptors at low frequencies [33], [34] and the frequency-dependent summation of DA release and uptake. At 6–14 months there was no significant difference in the frequency response between genotypes (normalised to peak [DA]_o_ evoked by a single pulse) (*n = 9–15*)(**Fig. 2b** **upper left**). At 18–24 months, normalised peak [DA]_o_ evoked by an intermediate frequency (10 Hz) was slightly increased in line B compared to WT (2-way ANOVA, *post hoc* Bonferroni t test, *P<0.01*; *n = 9–15*), but not in line A (**Fig. 2b** **upper right**).

The frequency dependence of striatal DA release is strongly controlled by acetylcholine (ACh) [31], [35]. ACh is released from striatal cholinergic interneurons and acts at nicotinic acetylcholine receptors (nAChRs) on DA axons. When nAChRs are active, initial DA release probability in response to a given depolarising stimulus is relatively high [31], [36]. DA release then shows strong short term depression so, as shown above, release is relatively insensitive to firing frequency. When nAChRs are inhibited, initial DA release probability is reduced. This reduction relieves depression during a train and release becomes strongly dependent on stimulation frequency [31], [35]. *In vivo*, striatal cholinergic interneurons show dynamic changes in activity, ranging from pauses to brief bursts that might co-vary with activity in DA neurons [37]–[39] but the state of nAChR activation is not known. In any event, nAChR activity will mask, or confound, the regulation of DA release by presynaptic mechanisms that are intrinsic to the DA axon. Therefore we also investigated whether overexpression of A53Tα-syn modifies DA release when nAChRs are inhibited, using the nAChR antagonist DHβE (1 µM).

With DHβE, [DA]_o_ evoked by 4 pulses, showed a strong frequency sensitivity, increasing with increasing frequency (**Fig. 2a,b** **lower**), as previously reported [31], [35]. This frequency response was seen in all genotypes across ages. However, at 6–14 months, peak [DA]_o_ evoked by 100 Hz stimulation (normalised to [DA]_o_ evoked by a single pulse) was lower in line B compared to WT (2-way ANOVA, *post hoc* Bonferroni t test, *P<0.001*; *n = 9–15*). There was no significant difference between line A and WT (**Fig. 2b** **lower left**). In contrast at 18–24 months, peak [DA]_o_ evoked by 100 Hz stimulation (normalised to [DA]_o_ evoked by a single pulse) was lower in both lines A and B compared to WT (2-way ANOVA, *post hoc* Bonferroni t test, *P<0.05*; *n = 9–15*)(**Fig. 2b** **lower right**).

### A53Tα-syn overexpression effects on re-release

**Figure 3:**
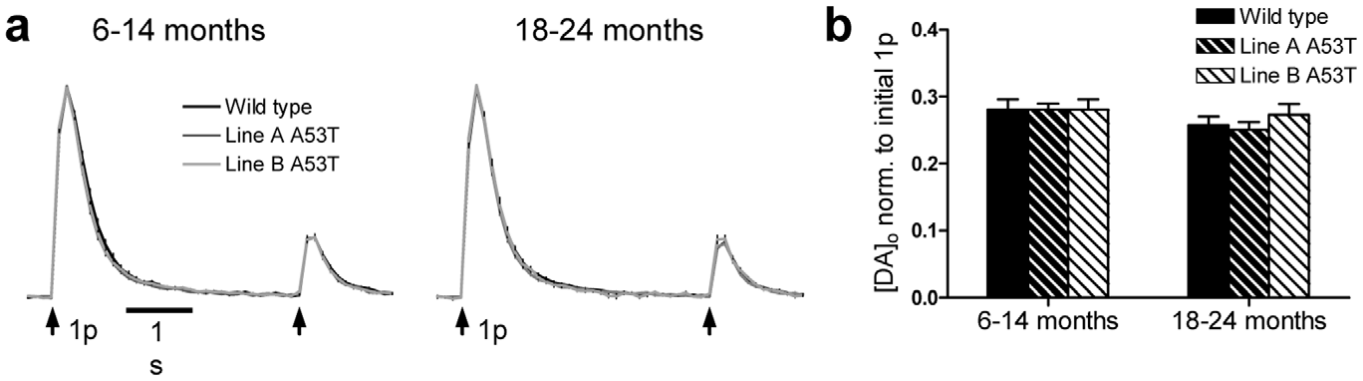
DA re-release following a single pulse stimulation is unchanged in A53Tα-syn overexpressors. (**a**) Profiles of mean [DA]_o_± SEM versus time, evoked by 2 single pulse stimulations, separated by 4 s. There was no significant difference in normalised [DA]_o_ evoked by a second pulse between genotypes, at either age, in either region (CPu only shown)(*n = 15–40*). (**b**) Mean peak [DA]_o_±SEM evoked by second stimulus pulse, normalised to peak [DA]_o_ evoked by first stimulus pulse.

We explored the effect of A53Tα-syn overexpression on the recovery of DA re-release evoked by single pulses in two paradigms, either following a single stimulus pulse or following a prolonged stimulus train. In the first protocol, re-release was explored at second pulse given at an inter-stimulus interval of 4 s, a timepoint when deletion of α-synuclein has been reported to modify DA re-release in some studies [40], although not others [41]. The ratio of peak [DA]_o_ evoked by this second compared to the first pulse was ∼0.3, in all genotypes at both ages (**Fig. 3**). This is characteristic of the strong short term depression of DA release seen *in vitro*, from which DA release takes 30–60 s to recover [25], [40]. There was however, no significant difference between genotypes in the P2/P1 ratio, for either age range, in CPu (**Fig. 3**, *n = 15–40*) or NAc (data not shown).

**Figure 4:**
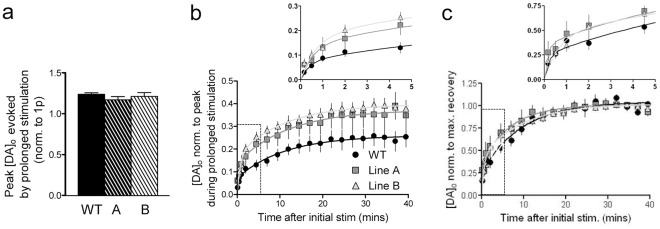
A53Tα-syn overexpressors show enhanced recovery of DA release following prolonged stimulation. (**a**) Mean peak [DA]_o_± SEM evoked during a prolonged 10 Hz train (60 s), normalised to mean peak [DA]_o_ evoked by a single pulse. There was no significant difference in normalised peak [DA]_o_ between genotypes (*n = 8–10*). (**b,c**) Mean peak [DA]_o_± SEM evoked by a single stimulus pulse, against time after the end of initial prolonged stimulation. Values were normalised to (**b**) peak [DA]_o_ evoked by initial 10 Hz train or to (**c**) maximum [DA]_o_ evoked by subsequent single pulses. Curve fits are two phase exponential associations, *R^2^>0.80*. Both sets of curves are significantly different between genotypes. Inset is the expanded initial 5 minutes following the end of prolonged stimulation, showing the same curve fits as main graphs.

It has been reported that α-syn may control recycling or reclustering of vesicle pools after more prolonged stimulation [15], [16]. Thus in the second protocol to explore re-release, we explored recovery of release after prolonged stimulation trains (60 s at 10 Hz) after which DA release is undetectable for several seconds before partial recovery. These experiments focussed on old-aged animals only (18–24 months) in CPu. These experiments were carried out in the presence of a mixture of antagonists for glutamate (NMDA: D-APV, 50 µM, mGluR: (S)-MCPG, 200 µM, AMPA: GYKI52466, 10 µM), GABA (GABA_A_: bicuculline, 10 µM, GABA_B_: saclofen, 50 µM) and D_2_ (L-714,626, 1 µM) receptors which may modulate DA release during long stimulation trains (but not significantly during sub-second stimulations [25], [42]). A prolonged 10 Hz stimulus train (60 s) was applied and DA re-release was monitored using a single stimulus pulse at timepoints 10, 30, 60, 120, 270 s and at subsequent 150 s intervals after the end of the 10 Hz train. There was no significant difference between genotypes in the mean peak [DA]_o_ evoked by the initial long 10 Hz stimulus train (**Fig. 4a**) but, recovery of single pulse [DA]_o_ varied with genotypes when normalised to either the peak [DA]_o_ evoked by the initial stimulus (**Fig. 4b**) or to the maximum recovery of [DA]_o_ (**Fig. 4c**). DA release by a single pulse recovered to ∼25–35% of release evoked by the initial prolonged stimulation, but to a significantly greater extent (**Fig. 4b**) and with a significantly enhanced rate (**Fig. 4c**) in both A53Tα-syn overexpressing lines compared to WT. Two-phase exponential curve fits of both data sets (*R^2^>0.80*) are significantly different between genotypes.

## Discussion

Mice that overexpress A53T-SNCA may be a useful model to explore the processes underlying PD since they have abnormalities in striatal presynaptic protein expression and postsynaptic signalling, and have cellular aggregations and motor behaviours that are suggestive of an age-related deficit in DA signalling [19]–[21]. Somewhat paradoxically, these mice have also been reported to have elevated striatal DA content [20]. DA releasability in this model has however previously remained unknown. Here, we explored directly the properties of DA release in the striatum of these mice using subsecond detection at extracellular carbon microelectrodes. We show that normal extracellular concentrations of DA can be evoked in these mice, but that A53Tα-syn overexpression causes subtle modifications to some properties of release. In particular, A53Tα-syn overexpressors showed a deficit in the frequency responsiveness of DA release, when the confounding effects of striatal ACh acting at presynaptic nAChRs were antagonized, and a slight increase in the recovery of DA release following prolonged stimulation, in old-aged mice. These changes in regulation of release are modest but may contribute to or indicate striatal dysfunction.

### Absolute [DA]_o_ is not modified

α-synuclein has been reported to be a negative regulator of transmitter release, in both dopaminergic and non-dopaminergic systems [11], [16], [28], [40], [41] and knockout of the synuclein family results in elevated [DA]_o_ evoked by single pulses in dorsal striatum, but not in the adjacent NAc [28]. We investigated whether conversely, overexpression of A53Tα-syn reduced single pulse-evoked DA release in CPu or NAc, but A53Tα-syn overexpression had no significant effect on mean peak DA release evoked by single stimuli, in either striatal region, in either mid-age (6–14 months) or old-age (18–24 months) mice. Analysis of the cumulative distribution of individual data points did indicate slight changes in DA releasability in a subset of sites in CPu, due either to a relative reduction in low releasing sites or increase in high-releasing sites, but overall, [DA]_o_ evoked in A53T overexpressors were typical of those seen in wild-types. However, these data, alongside the finding that A53Tα-syn overexpressors have a 20–30% increase in striatal DA content, indicate that there is a decrease in the fraction of stored DA that is released by each stimulus, and may thus reflect an underlying deficit in DA releasability.

### Frequency response of DA release is subtly modified

DA neurons fire at a range of frequencies *in vivo*, [26], [27] and DA release can be differently regulated or filtered during these different frequencies of activity [31], [35]. This filtering is strongly dependent on striatal network factors, including ACh released from striatal cholinergic interneurons, which acts at nAChRs on DA axons to dynamically modulate DA release [31], [35], [36]. Thus it was important here to assess [DA]_o_ evoked by a full range of physiologically relevant firing frequencies with and without activation of nAChRs. In drug-free conditions, when nAChRs can be activated by endogenous striatal ACh, the frequency response of DA release followed a characteristic inverted-U relationship in all genotypes [23]. The frequency response of DA release was broadly similar across genotypes but was subtly altered at intermediate frequencies in old-aged mice in one line of A53Tα-syn overexpressors, line B. This outcome is difficult to interpret since it was not common to both lines of A53T overexpressors, but may reflect a more pronounced phenotype in this line as evidenced by a higher degree of overexpression and earlier deficits in spontaneous locomotion in this line (see [19]).

When the confounding effects of nAChRs were removed, through addition of an nAChR antagonist, the underlying frequency sensitivity of DA release was enhanced in each genotype (as published previously in wild types [31], [35], [36]), but notably the overall frequency sensitivity of evoked [DA]_o_ was significantly less in A53Tα-syn overexpressors compared to wild-type, in one A53Tα-syn line of mid-aged mice (line B) and in both lines of old-aged mice. Reduced frequency sensitivity can arise through a number of mechanisms. In many neuronal systems, frequency sensitivity and initial transmitter release probability are inversely correlated [43], [44], since an increased release probability leads to increased short-term depression of release, and hence a decrease in subsequent transmitter release. Therefore reduced frequency sensitivity of DA release may result from an increase in initial release probability [25], [45]. Alternatively it may arise from a reduction in short-term facilitatory processes that underlie short-term plasticity in neurotransmitter release. Through either possible underlying mechanism, the dynamic increases in extracellular DA concentrations that are thought to signal bursts in DA neuron firing to postsynaptic neurons, may be compromised by this deficit, and in turn contribute to the progressive postsynaptic changes, such as increased DA receptor expression, and motor deficits that occur in these mice.

### Modified DA synapse function is indicated by changes to DA re-release

α-syn has been implicated in the control of transmitter vesicle pools, their trafficking, reclustering and re-release [13]–[16], [40]. In addition A53Tα-syn overexpressors show upregulation of several proteins associated with vesicle trafficking and cycling, such as the chaperone protein 14-3-3 and endocytic protein dynamin [21]. We used two different protocols differing in stimulation intensity, to explore whether A53T overexpression modified DA re-release in CPu. Release in a mild paired-pulse paradigm was not different between genotypes but following a prolonged stimulus train that was designed to deplete DA terminals of readily releasable vesicles [46], overexpression of A53Tα-syn altered the recovery of DA release in old-aged animals. The extent and rate of recovery of release was significantly greater in both lines of A53Tα-syn overexpressors compared to WT. These data are in keeping with the enhanced DA storage capability of these animals [20], but may also indicate an enhanced vesicle reserve pool which can more readily replace the normally releasable pool; indeed hippocampal neurons from α-syn knockout mice are reported conversely to have a decrease in the size of glutamate reserve pools [14]. Alternatively this enhanced DA re-releasability may result from the upregulation of vesicle chaperone proteins seen in these mice [21]. Then again, the increase in re-release could be caused by an upregulation of any number of mechanisms that contribute to DA availability (e.g. synthesis), and could thus be in keeping with compensatory presynaptic adaptations that have occurred *in vivo* after A53T expression to offset deficits in DA releasability e.g. those resulting from reduced fractional DA release or deficits in high frequency release. These data are however inconsistent with findings in cultured neurons that α-syn overexpression decreases vesicle reclustering, and hence transmitter release, following prolonged stimulation [16]. Surprisingly little is established regarding the factors that regulate the availability and releasability of vesicular pools for DA, and DA vesicles do not readily cluster anatomically at presynaptic active zones [28] unlike at synapses for many other transmitters e.g. glutamate [47], suggesting that different factors may regulate vesicular pools for DA compared to glutamate.

### Conclusions

Here we show that A53Tα-syn overexpressing mice have subtle modifications to striatal DA release. They have small but significant deficits in the sensitivity of DA release to frequency of activity, and they have enhanced recovery of DA re-release following prolonged stimulation. These DA synapse dysfunctions are modest but may nonetheless contribute to postsynaptic changes and motor deficits.
